# Thrombomodulin activation driven by LXR agonist attenuates renal injury in diabetic nephropathy

**DOI:** 10.3389/fmed.2022.916620

**Published:** 2023-01-09

**Authors:** Wei Wang, Song Wu, Amanda Y. Wang, Tao Wu, Haojun Luo, Jia Wei Zhao, Jin Chen, Yi Li, Hanlu Ding

**Affiliations:** ^1^Renal Division and Institute of Nephrology, Sichuan Academy of Medical Science & Sichuan Provincial People's Hospital, School of Medicine, University of Electronic Science and Technology of China, Chengdu, Sichuan, China; ^2^Renal and Metabolic Division, The George Institute for Global Health, University of New South Wales Australia, Newtown, NSW, Australia; ^3^Department of Renal Medicine, Concord Repatriation General Hospital, Concord Clinical School, University of Sydney, Camperdown, NSW, Australia; ^4^Faculty of Medicine, Health and Human Sciences, Macquarie University, Sydney, NSW, Australia; ^5^Internal Medicine, Louisiana State University Health Science at Shreveport, Shreveport, LA, United States; ^6^The Faculty of Health Sciences and Medicine, Bond University, Gold Coast, QLD, Australia

**Keywords:** liver X receptor, thrombomodulin, factor XIII-A, inflammation, diabetic nephropathy

## Abstract

**Objective:**

Inflammation and thrombosis are recognized as interrelated biological processes. Both thrombomodulin (TM) and factor XIII-A (FXIII-A) are involved in inflammation and coagulation process. However, their role in the pathogenesis of diabetic nephropathy (DN) remains unclear. *In vitro* study, the liver X receptor (LXR) agonist T0901317 can up-regulate the expression of TM in glomerular endothelial cells. Now we evaluated the interaction between TM activation and FXIII-A and their effects against renal injury.

**Methods:**

We first evaluated the serum levels of FXIII-A and TM and the expression of TM, LXR-α and FXIII-A in renal tissues of patients with biopsy-proven DN. We then analyzed the expression of TM, LXR-α and FXIII-A in renal tissues of db/db DN mice after upregulating TM expression *via* T0901317 or downregulating its expression *via* transfection of TM shRNA-loaded adenovirus. We also investigated the serum levels of Tumor necrosis factor (TNF)-α, Interleukin (IL)-6, creatinine, and urinary microalbumin level in db/db mice.

**Results:**

Our study showed that elevations in serum levels of FXIII-A positively correlated to the serum levels of TM and were also associated with end-stage kidney disease in patients with DN. The number of TM^+^ cells in the renal tissues of patients with DN negatively correlated with the number of FXIII-A^+^ cells and positively correlated with the number of LXR-α^+^ cells and estimated glomerular filtration rate (eGFR), whereas the number of FXIII-A^+^ cells negatively correlated with the eGFR.

**Conclusion:**

Thrombomodulin activation with T0901317 downregulated FXIII-A expression in the kidney tissue and alleviated renal injury in db/db mice.

## Introduction

Diabetic nephropathy (DN), as one of the leading causes of end stage kidney disease (ESKD), represents a heavy burden to people all over the world (1). Evidence suggests that inflammation plays a crucial role in the development of DN (2), characterized by oxidative stress and increased circulating inflammatory mediators (2–7). This accompanying inflammation leads to hypercoagulation and hypofibrinolysis, resulting in coagulopathies (4). Inflammation and thrombosis are increasingly recognized as interrelated biologic processes but their role in the pathogenesis of DN remains unclear (8, 9).

Factor XIII-A (FXIII-A) is a key factor involved in the final step in the formation of the fibrin network during coagulation (10). It is a transglutaminase that is composed of two A-subunits and two carrier B-subunits (FXIII-A2B2). The activation of FXIII by thrombin through cleavage of the activation peptide separates A and B subunits (11). FXIII-A is highly induced during the alternative activation of macrophages, while its expression is almost totally inhibited in response to stimuli inducing the classical activation pathway of coagulation (12). The selective expression of FXIII-A in M2 macrophages is in line with the capacity of this transglutaminase to act as an anti-inflammatory molecule (12). Thrombomodulin (TM) is involved in the clotting process and shows an anti-inflammatory effect in diabetic mice (13). TM is known to bind to thrombin on the endothelial cell surface and catalyzes activation of protein C, thus protecting cells from thrombus formation (13). Importantly, TM could influence FXIII-A activation by direct and indirect mechanisms (14). However, the role of TM interacting with FXIII-A against DN is largely unknown.

We recently revealed that activation of liver X receptor (LXR), by its agonist T0901317, can up-regulate the expression of TM in glomerular endothelial cells (15). T0901317 might inhibit the secretion of inflammatory mediators by regulation of competitive binding between NF-κB p65 and p300 (15). In this study, we evaluated the serum levels of FXIII-A and TM, the expression of TM, LXR-α and FXIII-A in the renal tissue of patients with type 2 diabetic nephropathy. In addition, we investigated the effects of T0901317 on TM activation upon renal inflammation and the expression of FXIII-A using db/db diabetic mice. We aimed to reveal the interaction between FXIII-A and TM activation and their protective effects against renal injury in db/db mice after up-regulating/down-regulating TM expression.

## Methods

### Patients

The diagnosis and classification of diabetes mellitus (DM) was based on the criteria of the American Diabetes Association (ADA) (16). DN was biopsy-proven diagnosis according to the standards of the Renal Pathology Society (RPS) in 2010 (16). Formalin-fixed, paraffin-embedded renal tissues obtained from core needle biopsies of patients with DN (*n* = 95) between 2014 and 2020 at Renal Division and Institute of Nephrology, Sichuan Provincial People's Hospital were included in this study. Subjects with primary kidney disease, secondary kidney disease due to other causes, superimposed systemic diseases, infection and blood system diseases were excluded. Patients with acute inflammatory disease, such as fever, urinary tract infection, pneumonia and sepsis, were also excluded. The other fifteen normal renal tissue specimen, taken from patients with traumatic renal injury or renal tumors, were used as the control group. Serum and 24-h urine specimen before renal biopsy were collected and preserved in a refrigerator at −80°C.

### Clinical characteristics

Clinical information was collected from electronic medical records at the time of the renal biopsy. An estimated glomerular filtration rate (e-GFR) was calculated using the creatinine-based Chronic Kidney Disease Epidemiology Collaboration equation (CKD-EPI). The level of serum fibrinogen was measured using the commercial assay kit based upon the clotting method of Von Clauss (17) with normal reference range of 1.8–3.5 g/L. The concentration of serum TM in DN patients and healthy control was measured by the ELISA. At the start of monitoring, the serum FXIII-A level was measured and patients with DN were divided into two groups: one with high (>658 pg/ml, mean + 2 SDs of healthy controls) FXIII-A level (*n* = 35, 777.63 ± 89.20 pg/ml) and another with normal FXIII-A level (<658 pg/ml; *n* = 60, 549.60 ± 87.66 pg/ml).

### Ad-TM shRNA construct

The 72 nt oligonucleotide encoding mouse TM shRNA was inserted into the BamHI and EcoRI sites of the shuttle plasmid pHBAd-U6-GFP to build the pHBAd-U6-GFP-TM shRNA plasmid. Then the shuttle plasmid pHBAd-U6-GFP-TM shRNA was checked through DNA sequencing after restriction enzyme treatment. Confocal and RT-PCR detected the expression of TM following the transfection of AD293 cells with pHBAd-U6-GFP-TM shRNA. A control adenovirus containing GFP tag was used as the Ad-Ctrl shRNA. It was similarly generated as pHBAd-U6-GFP- TM shRNA.

### Animals and drug treatment

Male db/db mice (C57BLKsJ background) aged from 6 to 8 weeks were obtained from the Junke Bioengineering Co., Nanjing, China. According to previous studies, db/db mice with blood glucose levels higher than 400 mg/dl for the ages between 6 and 8 weeks could be considered as diabetic mice (18). In this study, we observed that serum creatinine and urinary microalbumin levels significantly elevated in db/db mice aged 12 weeks when compared with wild type C57Bl/6 mice. Furthermore, the 12 weeks old db/db mice presented typical pathological changes of renal damage such as increased glomerular volume and extracellular matrix, and hyperplasia of mesangial cells. Thus, db/db mice aged 12 weeks demonstrated significant DN features. We set the observation time to 4 weeks for db/db mice aged 8–12 weeks. In our preliminary experiment, the wild type mice were treated with 2.5 × 10^9^ pfu and 1.25 × 10^9^ pfu Ad-TM shRNA by i.v. retrospectively. The wild type mice were also orally treated with 0.1, 0.25, and 0.5 mg/kg/day LXR agonist T0901317(#T2320, Sigma-Aldrich, St.Louis, USA). We found that 2.5 × 10^9^ pfu Ad-TM shRNA could lower the expression of TM, whereas 0.5 mg/kg/day T0901317 could increase the expression of TM. Therefore, we chose 2.5 × 10^9^ pfu Ad-TM shRNA injection and 0.5 mg/kg/day T0901317 gavage in this experiment. Twenty-four male db/db mice were randomly assigned to four groups (*n* = 6): diabetic control (diabetic ctrl) group, Ad-ctrl group, T0901317 group and Ad-TM shRNA group. For Ad-ctrl group, Ad-ctrl shRNA was intravenously injected into each db/db mouse in a single dose of 2.5 × 10^9^ PFU with volume at 50 μl. For Ad-TM shRNA group, Ad-TM shRNA was also intravenously injected into each db/db mouse in a single dose of 2.5 × 10^9^ PFU with volume at 50 μl. For T0901317 group, the db/db mice were orally treated with 0.5 mg/kg/day T0901317 once per day for 7 days. Male wild C57BL6 mice of the same age and DN control mice were given 50 μl of physiological saline by gastric perfusion once per day for 7 days. 4 weeks after Ad-TM shRNA injection, all the mice were euthanised, and all renal tissues, serum samples and urine samples were collected.

### ELISA detection

The concentrations of serum TM and FXIII-A in DN patients and healthy control were measured using the TM (ZC-M6306, Shanghai Zhuocai Biotechnology Co., Ltd., China), FXIII-A ELISA kit (ZC-33330, Shanghai Zhuocai Biotechnology Co., Ltd., China) as per the instructions of the manufacturer. The serum levels of TNF-α and IL-6 in murine were measured by TNF-α ELISA kit (CSB-E04741, Wuhan Huamei Company, China) and IL-6 ELISA kit (CSB-E04639, Wuhan Huamei Company, China) following the instructions offered by the manufacturer.

### Western blot assay

Samples from murine kidney tissues were lysed and quantified by Bradford assay. Twenty micrograms of protein samples were boiled at 100°C for 5 min after being mixed with an equal volume of 2×SDS sample buffer. The samples were separated by SDS-polyacrylamide gel electrophoresis and transferred to PVDF membranes (Thermofisher Scientific). The membranes were incubated with primary antibodies against β-actin (1:20,000), TM (1:1,000, ab109189, Abcam Company), LXR-α (1:1,000, ab176323, Abcam Company) and FXIII-A (1:10,000, 76105, Beijing Zhongshan Jinqiao biological Co., Ltd., China).

### Detection of immunohistochemical staining

For immunohistochemistry staining, the dewaxed and hydrated 2 μm renal tissue sections were incubated in 3% H_2_O_2_. The sections were washed with PBS (pH7.2–7.4) for 5 min, 3 times. After the sections were blocked with 5% BSA for 30 min, they were incubated with primary antibodies against TM (1:100, ab109189, Abcam Company), LXR-α (1:100, ab176323, Abcam Company) and FXIII-A (1:500, 76105, Beijing Zhongshan Jinqiao biological Co., Ltd., China). After wards, the sections were washed with PBS (pH7.2–7.4) 3 times for 5 min/time, and stained with Polink-2 plus Polymer HRP Detection System (PV-9001/9002, ZSGB-Bio, China). The histological sections were assessed by a certified pathologist without knowledge of the treatment groups.

### Immunohistochemical assessment of kidney

The staining intensity of TM in glomeruli or tubular interstitium can be divided into the following grades: 0, negative; 1+, positive in < 25% of capillary loop or interstitium; 2+, positive in 25–50% of capillary loop or interstitium; 3+, positive in 50–75% of capillary loop or interstitium and 4+, positive in 75% of capillary loop or interstitium. The average value of 10 glomerulus or tubular interstitium in each section was taken as the staining intensity of TM in the glomerulus or tubular interstitium of the patient, respectively. The staining intensity of LXR-α in the kidney tissue was divided into the following grades: 0, negative; 1, weakly positive; grade 2, moderately positive; grade 3, strongly positive. Ten fields were analyzed, and the results were expressed as the mean ± SD. The numbers of FXIII-A^+^ cells in the glomerular infiltrate were expressed as the numbers of positive cells per glomerulus. Scores of 0 = 0 positive cells, 1 = 1–4 cells, 2 = 5–10 cells, and 3 > 10 cells were applied. The numbers of FXIII-A^+^ cells in tubular interstitium were semi-quantitatively evaluated on a scale from 0 to 4+ (0, absent staining; 1+, moderately strong staining in < 25% of cells; 2+, strong staining in 25–50% of cells; 3+, very strong staining in 50–75% of cells; and 4+, very strong staining in 75% of cells). Histopathological evaluation was performed by an investigator who was not aware of the data (19).

### Statistical analysis

Statistical analysis was performed with the SPSS software system (SPSS for Windows, version 25.0; SPSS Inc., Chicago, IL). Parametric data were statistically analyzed by one-way ANOVA. Differences in non-parametric data were evaluated by the Mann-Whitney *U*-test. Correlation analysis was conducted using the Spearman test for nonparametric data. Data were expressed as means ± SD. A statistically significant difference was defined as *p* < 0.05.

## Results

### Characteristics of DN patients at the time of renal biopsy

Ninety five patients enrolled in this study, their baseline characteristics were shown in Table 1. The serum levels of TM was 4.89 ± 0.91 ng/ml in normal serum FXIII-A level group and 7.62 ± 1.48 ng/ml in high serum FXIII-A level group. During the follow-up 1 year later, the percentage of patients who progressed to end-stage kidney disease (ESKD) from baseline was 16.7% in normal FXIII-A group and 40.0% in high FXIII-A group (Table 1).

**Table 1:** Clinical features of DN patients with different serum FXIII-A levels at baseline.

**Parameter**	**High FXIII-A group** **(*n* = 35)**	**Normal FXIII-A group** **(*n* = 60)**	***P*-value**
Age (years)	52.2 ± 6.96	54.07 ± 9.16	0.300
Gender (M/F)	26/9	46/14	0.808
FXIII-A (pg/ml)	777.63 ± 89.20	549.60 ± 87.66	0.000
TM (ng/ml)	7.62 ± 1.48	4.89 ± 0.91	0.000
Fibrinogen (g/l)	4.69 ± 1.17	4.05 ± 1.18	0.012
Diabetes duration (Months)	103.54 ± 61.95	108.13 ± 72.08	0.754
Diabetic retinopathy (%)	42.3(15)	38.3(23)	0.671
Hemoglobin (g/l)	108.17 ± 17.57	118.55 ± 20.27	0.013
BMI (kg/m^2^)	25.10 ± 2.74	24.56 ± 3.35	0.417
Smoking (%)	57.1(20)	43.3(26)	0.209
SBP (mmHg)	150.37 ± 24.71	146.63 ± 24.93	0.481
DBP (mmHg)	85.09 ± 12.24	83.57 ± 12.00	0.556
Serum creatine (mg/dl)	142.11 ± 53.32	110.44 ± 38.17	0.001
eGFR (>15 ml/min/1.73 m^2^)	55.63 ± 30.76	70.98 ± 30.30	0.020
Initial proteinuria (g/d)	5.44 ± 3.10	4.63 ± 3.97	0.302
HbA1c (%)	7.91 ± 2.66	8.14 ± 2.24	0.648
Total cholesterol (mmol/l)	2.59 ± 2.19	2.18 ± 1.62	0.295
Triglyceride (mmol/l)	3.12 ± 1.14	2.86 ± 1.26	0.318
RAAS inhibitor (%)	4/31	11/49	0.561
Progressed to ESKD (%)	40	16.7	0.021

Data are presented as the mean ± standard SBP, Systolic blood pressure; DBP, Diastolic blood pressure; e-GFR, estimated glomerular filtration rate; BMI, Body mass index; HbA1c, glycosylated hemoglobin; RAAS, Renin-angiotensin-aldosterone system; ESKD, end-stage kidney disease.

### Clinical features associated with high level of FXIII-A

**Figure 1:**
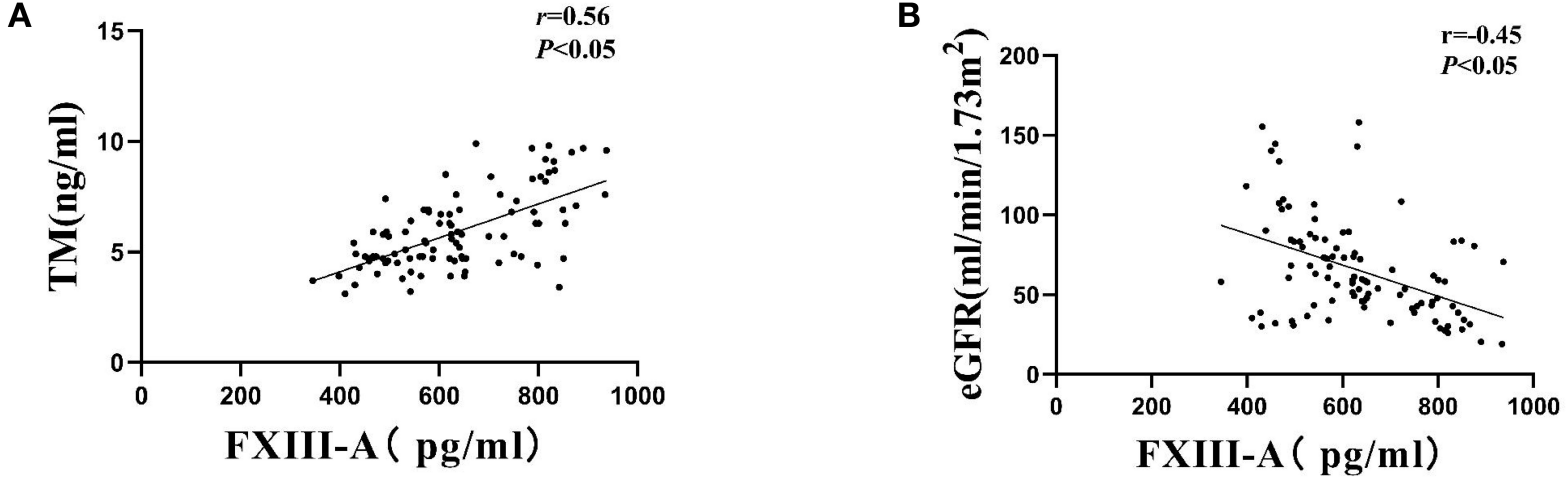
Correlations of serum FXIII-A levels with **(A)** serum TM levels and **(B)** eGFR among patients with DN.

The plasm FXIII-A level and TM level were 410.2 ± 124.02 pg/ml and 3.12 ± 0.68 ng/ml in healthy control, respectively. The plasm FXIII-A level and TM level were 633.61 ± 141.17 pg/ml and 5.90 ± 1.75 ng/ml in DN patients, respectively. Both the expression level of plasm FXIII-A and TM in DN patients was significantly higher than that of healthy control (*p* < 0.01). Furthermore, a higher serum TM, creatinine, fibrinogen level, lower e-GFR and hemoglobin level were observed in higher FXIII-A level compared with normal FXIII-A level groups (*p* < 0.05). However, there were no significant associations of serum FXIII-A concentration with age, duration of diabetes, diabetic retinopathy, body mass index (BMI), HbA1c, serum total cholesterol and triglycerides level, amounts of proteinuria, serum albumin level and percentage of hypertension and renin-angiotensinaldosterone system (RAAS) inhibitor use (Table 1). Moreover, the serum FXIII-A level had significant positive correlations with serum TM (*r* = 0.56, *p* < 0.01 Figure 1A), but a negative correlation with e-GFR (*r* = −0.45, *p* < 0.01; Figure 1B).

### The expression of TM, LXR-α, and FXIII-A in renal tissue of DN patients

**Figure 2:**
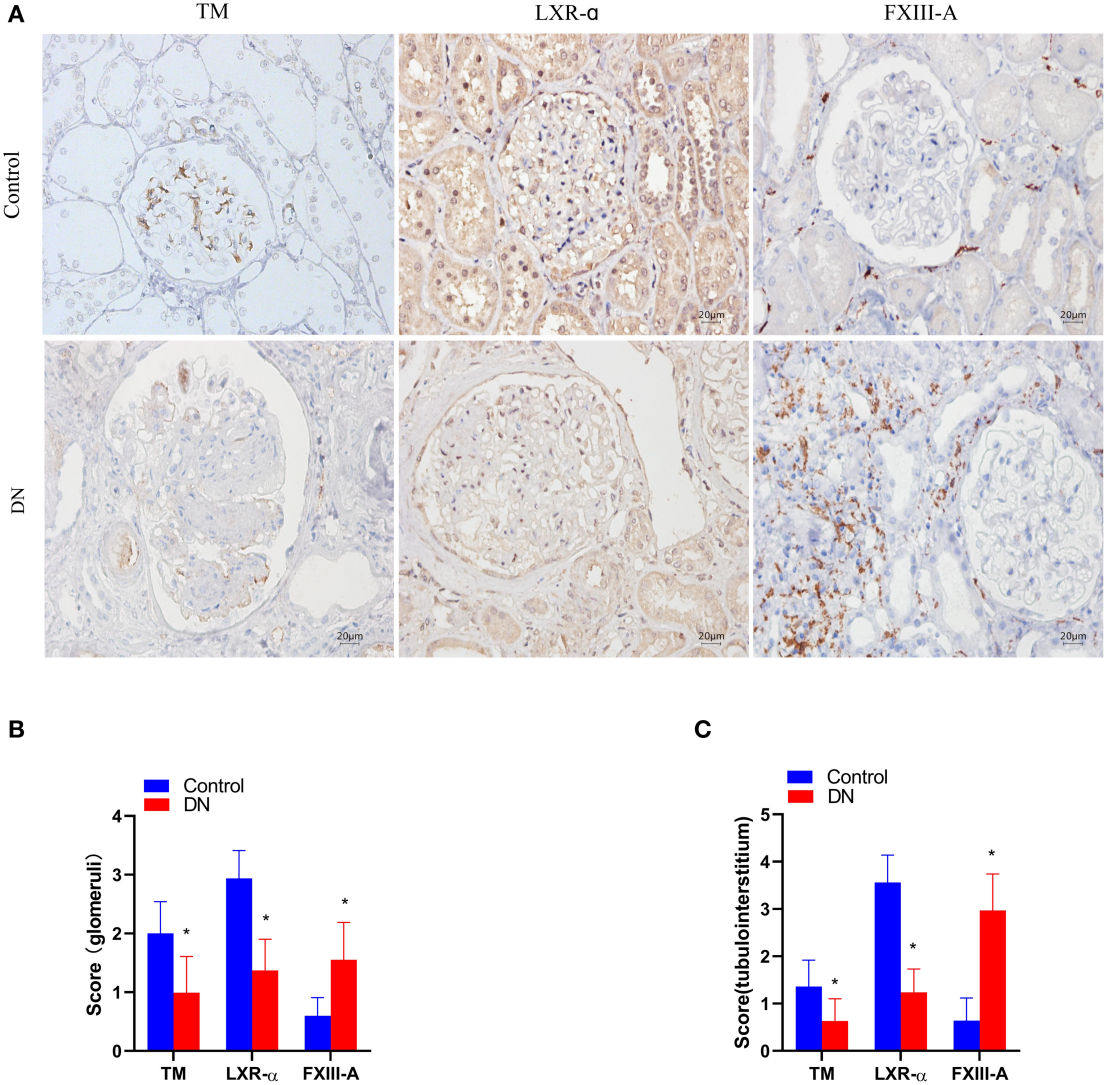
The expression of TM, LXR-α and FXIII-A in the renal tissue of DN patients. **(A)** Representative images of immunohistochemical staining of TM, LXR-α, and FXIII-A. Scale bar = 20 μm. **(B, C)** Score of positive staining for TM, LXR-α and FXIII-A in the kidney tissue (*n* = 15, mean ± SEM). *p < 0.05 compared with the Control group.

TM^+^ cells predominated at sites in glomerular capillaries and peritubular capillaries (Figure 1A). LXR-α^+^ cells were mainly observed in renal tubular epithelial cells and glomerular cells (Figure 2A). FXIII-A^+^ cells were occasionally expressed in normal kidney tissue, but there were numerous FXIII-A^+^ cells in glomeruli and tubulointerstitial tissue of DN patients (Figure 1A). The numbers of TM^+^ cells and LXR-α^+^ were decreased, whilst the number of FXIII-A^+^ cells was increased in the kidneys of DN patients when compared with that in healthy kidneys (Figures 2B, C). In the negative controls, TM, LXR-α and FXIII-A staining was all negative.

### Correlation of the number of TM^+^, LXR-α^+^, and FXIII-A^+^ cells in kidney tissue with 24 h urinary protein quantification and eGFR values in DN patients

The number of TM^+^ cells, LXR-α^+^ cells and FXIII-A+ cells in DN kidney tissue had no correlation with 24 h urinary protein excretion. The eGFR level at the time of renal biopsy showed positive correlations with the TM^+^ cells and LXR-a^+^ cells scores, but a negative correlation with the FXIII-A^+^ cells scores both in the glomerular and in the tubulointerstitial tissue (Table 2).

**Table 2:** Correlation analysis between TM+, LXR-α+, FXIII-A+ cells with eGFR level in DN patients.

**Parameter**	**eGFR (ml/mim/1.73 m** ^**2**^ **)**	
	***r***	***p***
**Glomerulus**		
TM^+^ cells	0.88	0.00
LXR-α^+^ cells	0.91	0.00
FXIII-A^+^ cells	−0.85	0.00
**Tubulointerstitium**		
TM^+^ cells	0.76	0.00
LXR-α^+^ cells	0.94	0.00
FXIII-A^+^ cells	−0.90	0.00

### The expression of TM, LXR-α, and FXIII-A in db/db mice

**Figure 3:**
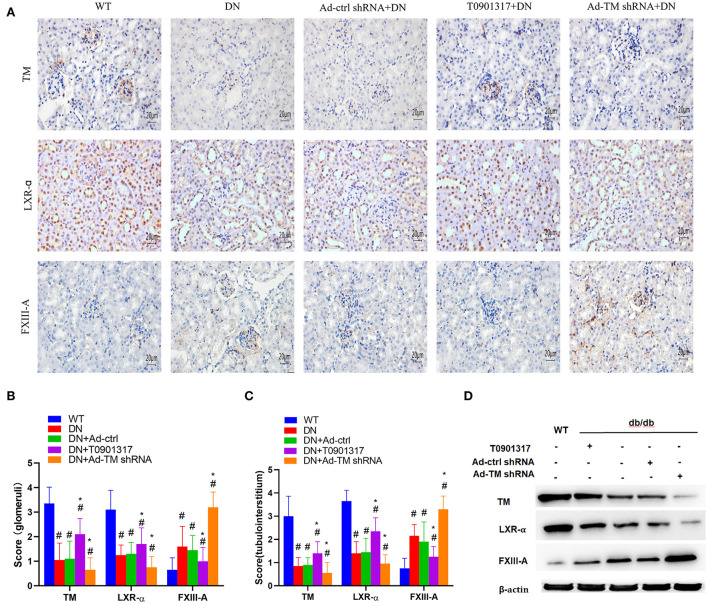
The expression of TM, LXR-α and FXIII-A detected by immunohistochemical staining and western blot in db/db mice and wild mice. db/db mice were divided into four groups: DN; Ad-ctrl+DN; T0901317+DN and Ad-TM shRNA+DN. The wild mice were as control. **(A)** Representative images of immunohistochemical staining of TM, LXR-α and FXIII-A. Scale bar = 20 μm. **(B, C)** Score of positive staining for TM, LXR-α and FXIII-A in the kidney tissue (*n* = 6, mean ± SEM). *p < 0.05 compared with the WT group. ^#^*p* < 0.05 compared with the DN group and Ad-ctrl+DN group. **(D)** The expression of TM, LXR-α and FXIII-A detected by western blot in db/db mice and wild mice.

TM^+^ cells were mainly observed in glomerular capillaries and peritubular capillaries, while LXR-α^+^ cells were mainly observed in renal tubular epithelial cell and glomerular cells by immunoperoxidase staining (Figure 3A). FXIII-A was occasionally expressed in normal kidney tissue, but there were numerous FXIII-A^+^ cells both in glomeruli and in tubulointerstitial tissue of db/db mice (Figure 3A). Compared with wild mice, the expression of FXIII-A^+^ cells was increased in both the db/db Ctrl group and the db/db Ad-Ctrl group. Compared with db/db mice, Ad-TM shRNA increased the expression of FXIII-A in renal tissue of db/db mice. However, T0901317 inhibited the positive expression of FXIII-A in renal tissue of db/db mice (Figures 3B, C). Both immunoperoxidase staining and western blotting analysis revealed that TM and LXR-α protein expression in the kidney was significantly downregulated in the db/db ctrl group and db/db Ad-ctrl group compared with the mild mice group (Figures 3B–D). T0901317 increased the protein expression of TM and LXR-α and decreased the FXIII-A protein expression in the kidney of db/db mice (Figures 3B–D).

### T0901317 inhibited inflammation in db/db mice

**Figure 4:**
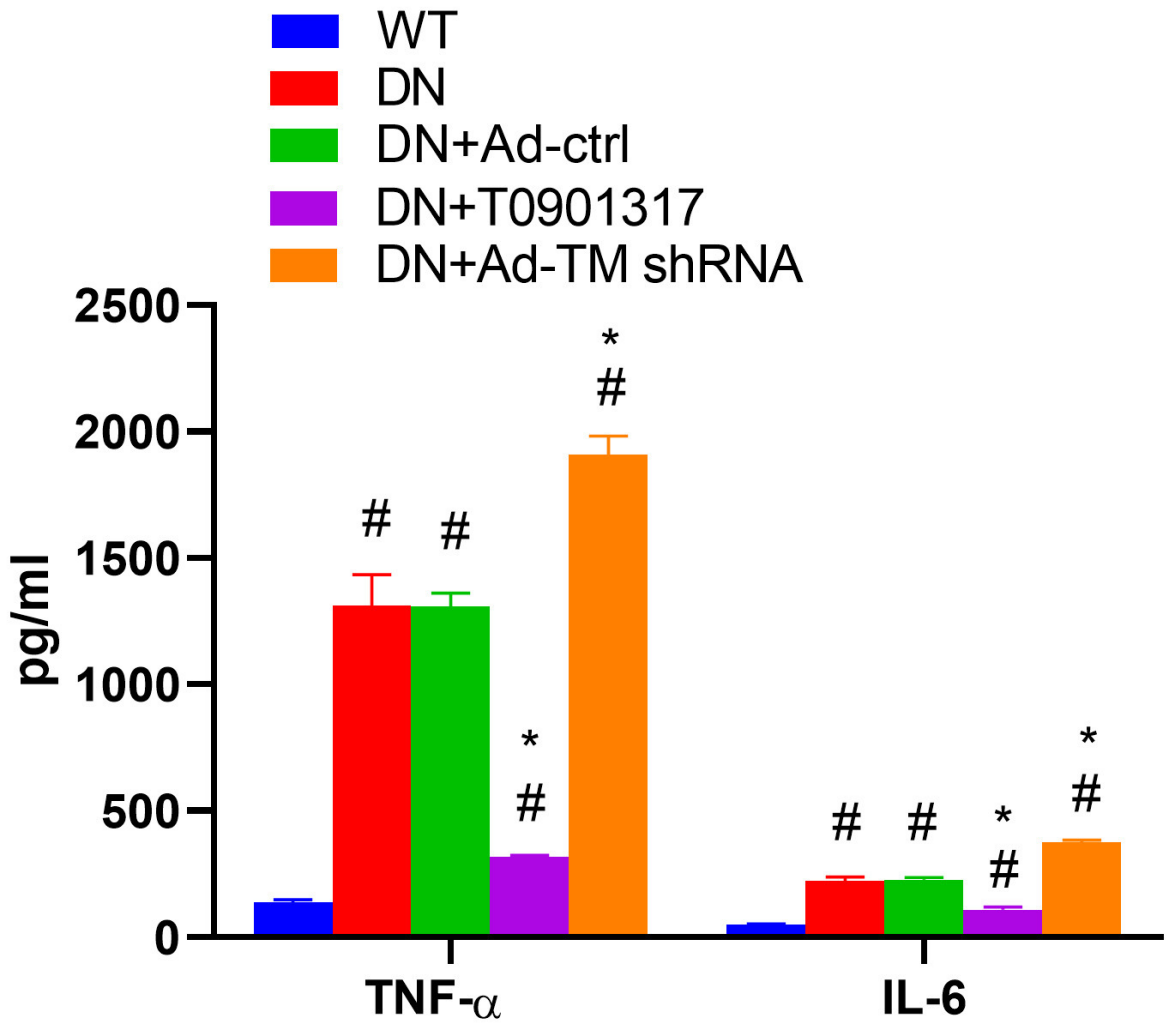
The level of serum TNF-α and serum IL-6 in db/db mice and wild mice (*n* = 6, mean ± SEM). *p < 0.05 compared with the WT group. ^#^*p* < 0.05 compared with the DN group and Ad-ctrl+DN group.

Compared with the wild mice, the serum TNF-α and serum IL-6 level was increased both in db/db Ctrl group and db/db Ad-Ctrl group. Ad-TM shRNA increased the serum TNF-α and IL-6 level. However, T0901317 inhibited the serum TNF-α and IL-6 level (Figure 4).

### LXR agonist T0901317 attenuated renal injury in db/db mice

Compared with the wild mice, urinary microalbumin and serum creatinine (Scr) level was increased both in db/db Ctrl group and db/db Ad-Ctrl group. Ad-TM shRNA injection increased the urinary microalbumin and serum creatinine level, whereas T0901317 administration attenuated urinary microalbumin and serum creatinine level in db/db mice (Table 3).

**Table 3:** LXR agonist T0901317 improves renal injury in db/db mice (*n* = 6, $\bar{x}$ ± s).

**Group**	**Scr (μmol·L^−1^)**	**Microalbuminuria** **(mg/L)**
Ctrl	25.17 ± 2.86	0.65 ± 0.027
DN	45.50 ± 1.88^*^	1.24 ± 0.11^*^
Ad-ctrl+DN	44.83 ± 3.06^*^	1.27 ± 0.02^*^
T0901317+DN	30.67 ± 2.16^*^^#^	0.72 ± 0.02^*^^#^
Ad-TMshRNA+DN	65.50 ± 2.90^*^^#^	1.88 ± 0.03^*^^#^

* *p* < 0.05, vs. Ctrl group;

# *p* < 0.05 vs. DN group and Ad-ctrl+DN group.

## Discussion

Inflammation and thrombosis are increasingly recognized as interrelated biological processes but their role in the pathogenesis of DN is still unclear (8, 9). Due to the important role of TM and FXIII-A in inflammation and coagulation process, we aimed to reveal the interaction between FXIII-A and TM activation and their effects to against DN.

First, the association between FXIII-A, TM, clinical features and renal prognosis in patients with T2DM and biopsy-proven DN was explored. Our results indicated that the elevated serum FXIII-A level was positively correlated to the serum TM level. During the follow-up 1 year later, the percentage of patients who progressed to ESKD from baseline was 16.7% in normal FXIII-A group and 40.0% in high FXIII-A group. These results indicated that the elevated serum FXIII-A level maybe associated with progression to ESKD in patients with DN. The number of TM^+^ cells in the kidney tissue of DN patients was negatively correlated with the number of FXIII-A^+^ cells, but positively correlated to eGFR, while the number of FXIII-A^+^ cells was negatively correlated to their eGFR. Our study indicated that FXIII-A may interact with TM and participate in the pathogenesis of DN. A previous study identified a possible connection between FXIII-A and type 2 diabetes in a mouse model with obesity-induced chronic low-grade inflammation (20). TM could influence FXIII activation by a direct and indirect mechanism (14, 15). It competes with fibrin for the binding of exosite 1 in thrombin. As the binding of fibrin to certain amino acid side-chains present in exosite 1 is essential to its cofactor activity on FXIII-A truncation by thrombin, TM inhibits the acceleration of FXIII activation on the surface of fibrin (14). An additional indirect mechanism, by which TM influences FXIII activation, operates through enhancing the thrombin-induced activation of protein C. Activated protein C proteolytically cleaves FVa and FXIII-A and down-regulates the promotion of thrombin generation by these activated clotting factors. Then, impaired thrombin generation might lead to decreased/delayed activation of FXIII (14). In this study, the expression of FXIII-A in the kidney tissue was decreased after TM knockdown in db/db mice and the number of TM^+^ cells in the kidney of DN patients was negatively correlated with the number of FXIII-A^+^ cells. Our findings suggest that TM may interact with FXIII-A in DN.

In the presence of Ca^2+^, the truncated FXIIIA2B2 dimer dissociates from the inhibitory B subunits and becomes an active transglutaminase (FXIII-A) (21). FXIII-A acts in the end stages of blood clotting to cross-link and stabilize deposited fibrin. FXIII-A also has novel implications in multiple processes (18, 22), including bacterial entrapment (23), wound healing through effects on angiogenesis (24–26), tightening of the endothelial barrier (27, 28), potential regulation of immune functions like phagocytosis, migration and survival (29). According to previous reports, the selective expression of FXIII-A in M2 macrophages is in line with the capacity of this transglutaminase to act as an anti-inflammatory molecule (28). However in our study, the number of FXIII-A^+^ cells were found negatively correlated to eGFR in DN patients. Ad-TM shRNA increased the expression of FXIII-A^+^ cells in renal tissue of db/db mice. These findings indicate that renal tissue expressed FXIII-A is in line with the degree of renal injury. As FXIII-A is a key factor that is involved in the final step in the formation of fibrin network during coagulation cascade (11), an increase in cellular FXIII-A levels may impact deposition of fibrin in the extracellular spaces and hence accelerate kidney injury in DN.

Secondly, the impact of TM activation on FXIII-A expression, inflammatory response and renal injury in db/db mice was assessed. TM expression was up-regulated with LXR agonist T0901317 and down-regulated by Ad-TM shRNA in db/db mice. Our study showed that T0901317 decreased the FXIII-A expression in the kidney, and decreased the serum TNF-α and IL-6 levels. T0901317 also decreased urinary microalbumin and serum creatinine levels in db/db mice. Ad-TM shRNA injection aggravated the FXIII-A expression and inflammation in the kidneys in db/db mice, and pathologically intensified the degree of renal injury in db/db mice. Collectively, these data suggest that TM activation driven by LXR agonist attenuates renal inflammation and improves kidney injury in DN.

TM mainly expressed in the membrane of endothelial cells, protects cells from thrombus formation and activates protein C synthesis against inflammation (30). Up-regulation of TM might be a potential therapy for microvascular diseases induced by inflammation in DN (31). Previous studies demonstrated that naturally abundant compounds might regulate TM hence protect endothelial cells against inflammation, but it did not work out in DN (32, 33). There are two main members in LXR family, LXR-α, and LXR-β. LXR-α always exists in cholesterol metabolizing tissues including kidney, liver, macrophages and fat. LXR-β often widely exists in various organs and tissues (34). In a previous study, we have revealed that LXR agonist T0901317 up-regulates the expression of TM in glomerular endothelial cells and reduces the secretion of inflammatory mediators *in vitro* (15). In this study, we found that LXR-α^+^ cells were mainly observed in renal tubular epithelial cell and glomerular cells both in DN patients and db/db mice. TM-positive cells were mainly observed in glomerular capillaries and peritubular capillaries both in DN patients and db/db mice. The expression sites of TM and LXR were close to each other, suggesting that they may interact with each other *in vivo*. Our study demostrated that LXR agonist T0901317 increased the expression of TM in the renal tissue of db/db mice. These findings indicate that T0901317 up-regulates TM expression *in vivo*. As the T0901317 increased TM expression, and the TM promoter analyses did not reveal the presence of any putative LXR response elements, the inhibition of TM transcription by the LXR agonist T0901317 may involve an indirect mechanism through the regulation of other transcription factors that support TM transcription.

## The limitations and strengths of the study

To our knowledge, this is the first study assessing the interaction between FXIII-A and TM activation and their protective effects against renal injury in db/db mice after up-regulating/down-regulating TM expression. However, this study has several limitations. Firstly, this study is a retrospective study with a limited sample size, selection bias and potential confounders may exist, which could have impacts on study results. Secondly, the serum FXIII-A, TM and fibrinogen levels were only measured once at baseline, sequential measurements during the follow up would help to further investigate their associations with ESKD due to DN. Thirdly, potential therapeutic interventions (such as the hypoglycaemic agents with renoprotective effects) during follow-up were not adjusted, which may confound the results.

In summary, this study adds the current literature by providing evidence that serum FXIII-A is associated with TM, and a high FXIII-A level maybe an risk factor for DN progression to ESKD in patients with T2DM. TM activation driven by LXR-α agonist protects against renal injury in db/db mice and is associated with down-regulated FXIII-A expression and decreased levels of inflammatory mediators in db/db mice. Our results suggest that FXIII-A and TM, which are involved in coagulation and inflammation process, may interact with and participate in the pathogenesis of diabetic nephropathy. These findings provide experimental evidence to target interactions between FXIII-A and TM activation as a potential therapeutic strategy against DN.

## Data availability statement

The raw data supporting the conclusions of this article will be made available by the authors, without undue reservation.

## Ethics statement

The studies involving human participants were reviewed and approved by the Ethics Committee for Clinical Research of Sichuan Academy of Medical Science & Sichuan Provincial People's Hospital. The patients/participants provided their written informed consent to participate in this study. Written informed consent was obtained from the individual(s) for the publication of any potentially identifiable images or data included in this article.

## Author contributions

All authors listed have made a substantial, direct, and intellectual contribution to the work and approved it for publication.

## References

[1] Anders HJ Huber TB Isermann B Schiffer M. CKD in diabetes: diabetic kidney disease versus nondiabetic kidney disease. Nat Rev Nephrol. (2018) 14:361–7. 10.1038/s41581-018-0001-y 29654297

[2] Rayego-Mateos S Morgado-Pascual JL Opazo-Ríos L Guerrero-Hue M García-Caballero C Vázquez-Carballo C. Pathogenic pathways and therapeutic approaches targeting inflammation in diabetic nephropathy. Int J Mol Sci. (2020) 21:3798–841. 10.3390/ijms21113798 32471207 PMC7312633

[3] Calle P Hotter G. Macrophage phenotype and fibrosis in diabetic nephropathy. Int J Mol Sci. (2020) 21:2806–20. 10.3390/ijms21082806 32316547 PMC7215738

[4] Sun J Liu C. Correlation of vascular endothelial function and coagulation factors with renal function and inflammatory factors in patients with diabetic nephropathy. Exp Ther Med. (2018) 16:4167–71. 10.3892/etm.2018.6718 30402157 PMC6200961

[5] Loretelli C Rocchio F D'Addio F Nasr MB Castillo-Leon E Dellepiane S. The IL-8-CXCR1/2 axis contributes to diabetic kidney disease. Metabolism. (2021) 121:154804. 10.1016/j.metabol.2021.154804 34097917

[6] Fiorina P Vergani A Bassi R Niewczas MA Altintas MM Pezzolesi MG. Role of podocyte B7-1 in diabetic nephropathy. J Am Soc Nephrol. (2014) 25:1415–29. 10.1681/ASN.2013050518 24676639 PMC4073425

[7] Solini A Usuelli V Fiorina P. The dark side of extracellular ATP in kidney diseases. J Am Soc Nephrol. (2015) 26:1007–16. 10.1681/ASN.2014070721 25452669 PMC4413770

[8] Oe Y Hayashi S Fushima T Sato E Kisu K Sato H. Coagulation factor Xa and protease-activated receptor 2 as novel therapeutic targets for diabetic nephropathy. Arterioscler Thromb Vasc Biol. (2016) 36:1525–33. 10.1161/ATVBAHA.116.307883 27283743

[9] Hamedani NS Biswas A Rudan O Tönges R Meyring C Tolle F. Functional and structural characterization of nucleic acid ligands that bind to activated coagulation factor XIII. J Clin Med. (2021) 10:677–92. 10.3390/jcm10040677 33578732 PMC7916480

[10] Hurják B Kovács Z Dönczo B Katona É Haramura G Erdélyi F. N-glycosylation of blood coagulation factor XIII subunit B and its functional consequence. J Thromb Haemost. (2020) 18:1302–9. 10.1111/jth.14792 32168410

[11] Soendergaard C Kvist PH Seidelin JB Pelzer H Nielsen OH. Systemic and intestinal levels of factor XIII-A: the impact of inflammation on expression in macrophage subtypes. J Gastroenterol. (2016) 51:796–807. 10.1007/s00535-015-1152-2 26660730

[12] Matsumoto K Yano Y Gabazza EC Araki R Bruno NE Suematsu M. Inverse correlation between activated protein C generation and carotid atherosclerosis in Type 2 diabetic patients. Diabet Med. (2007) 24:1322–8. 10.1111/j.1464-5491.2007.02289.x 17971179

[13] Zabczyk M Natorska J Undas A. Factor XIII and fibrin clot properties in acute venous thromboembolism. Int J Mol Sci. (2021) 22:1607–17. 10.3390/ijms22041607 33562624 PMC7914915

[14] Philippou H Rance J Myles T Hall SW Ariens RA Grant PJ. Roles of low specifificity and cofactor interaction sites on thrombin during factor XIII activation. Competition for cofactor sites on thrombin determines its fate. J Biol Chem. (2003) 278:32020–6. 10.1074/jbc.M305364200 12794066

[15] Ding H Li Y Feng Y Chen J Zhong X Wang N. LXR agonist T0901317 upregulates thrombomodulin expression in glomerular endothelial cells by inhibition of nuclear factor kappaB. Mol Med Rep. (2016) 13:4888–96. 10.3892/mmr.2016.5138 27082844

[16] Zhang J Wang Y Zhang R Li H Han Q Wu Y. Serum fibrinogen predicts diabetic ESRD in patients with type 2 diabetes mellitus. Diabetes Res Clin Pract. (2018) 141:1–9. 10.1016/j.diabres.2018.04.025 29684616

[17] Tervaert TW Mooyaart AL Amann K Cohen AH Cook HT Drachenberg CB. Pathologic classification of diabetic nephropathy. J Am Soc Nephrol. (2010) 21:556–63. 10.1681/ASN.2010010010 20167701

[18] Mitchell JL Mutch NJ. Let's cross-link: diverse functions of the promiscuous cellular transglutaminase factor XIII-A. J Thromb Haemost. (2019)17:19–30. 10.1111/jth.14348 30489000

[19] Li J Yu YF Liu CH Wang CM. Significance of M2 macrophages in glomerulonephritis with crescents. Pathol Res Pract. (2017) 213:1215–20. 10.1016/j.prp.2017.04.011 28554749

[20] Phasha MA Soma P Pretorius E Phulukdaree A. Coagulopathy in Type 2 diabetes mellitus: pathological mechanisms and the role of factor xiii-a single nucleotide polymorphisms. Curr Diabetes Rev. (2019) 15:446–55. 10.2174/1573399815666190130113328 30706822

[21] Li B Kohler HP Schroeder V. Identification of amino acid residues that are crucial for FXIII-A intersubunit interactions and stability. Blood. (2020) 135:145–52. 10.1182/blood.2019002127 31697820

[22] Dickneite G Herwald H Korte W Allanore Y Denton CP Cerinic MM. Coagulation factor XIII: a multifunctional transglutaminase with clinical potential in a range of conditions. Thromb Haemost. (2015) 113:686–97. 10.1160/TH14-07-0625 25652913

[23] Loof TG Mörgelin M Johansson L Oehmcke S Olin AI Dickneite G. Coagulation, an ancestral serine protease cascade, exerts a novel function in early immune defense. Blood. (2011) 118:2589–98. 10.1182/blood-2011-02-337568 21613262

[24] Soendergaard C Kvist PH Seidelin JB Nielsen OH. Tissue-regenerating functions of coagulation factor XIII. J Thromb Haemost. (2013) 11:806–16. 10.1111/jth.12169 23406195

[25] Shi DY Wang SJ. Advances of coagulation factor XIII. Chin Med J (Engl). (2017) 130:219–23. 10.4103/0366-6999.198007 28091415 PMC5282680

[26] Zaets SB Xu DZ Lu Q Feketova E Berezina TL Gruda M. Recombinant factor XIII diminishes multiple organ dysfunction in rats caused by gut ischemiareperfusion injury. Shock. (2009) 31:621–26. 10.1097/SHK.0b013e31818bbe21 18948851 PMC3024715

[27] Zaets SB Xu DZ Lu Q Feketova E Berezina TL Malinina IV. Recombinant factor XIII mitigates hemorrhagic shock-induced organ dysfunction. J Surg Res. (2011) 166:e135–42. 10.1016/j.jss.2010.12.001 21276979 PMC3269945

[28] Dull K Fazekas F Törocsik D. Factor XIII-A in Diseases: role beyond blood coagulation. Int J Mol Sci. (2021) 22:1459. 10.3390/ijms22031459 33535700 PMC7867190

[29] Yang SM Ka SM Wu HL Yeh YC Kuo CH Hua KF. Thrombomodulin domain 1 ameliorates diabetic nephropathy in mice *via* anti-NF-kappaB/NLRP3 inflammasome-mediated inflammation, enhancement of NRF2 antioxidant activity and inhibition of apoptosis. Diabetologia. (2014) 57:424–34. 10.1007/s00125-013-3115-6 24317792

[30] Loghmani H Conway EM. Exploring traditional and nontraditional roles for thrombomodulin. Blood. (2018) 132:148–58. 10.1182/blood-2017-12-768994 29866818

[31] He X Xu Z Wang B Zheng Y Gong W Huang G. Upregulation of thrombomodulin expression by activation of farnesoid X receptor in vascular endothelial cells. Eur J Pharmacol. (2013) 718:283–9. 10.1016/j.ejphar.2013.08.020 24041925

[32] Giri H Panicker SR Cai X Biswas I Weiler H Rezaie AR. Thrombomodulin is essential for maintaining quiescence in vascular endothelial cells. Proc Natl Acad Sci USA. (2021) 118:e2022248118. 10.1073/pnas.2022248118 33836597 PMC7980409

[33] van Aanhold CCL Dijkstra KL Bos M Wolterbeek R van den Berg BM Bruijn JA. Reduced glomerular endothelial thrombomodulin is associated with glomerular macrophage infiltration in diabetic nephropathy. Am J Pathol. (2021) 191:829–37. 10.1016/j.ajpath.2021.02.002 33617784

[34] Sohrabi Y Sonntag GVH Braun LC Lagache SMM Liebmann M Klotz L. LXR activation induces a proinflammatory trained innate immunity-phenotype in human monocytes. Front Immunol. (2020) 11:353–67. 10.3389/fimmu.2020.00353 32210962 PMC7077358

